# Flashbulb memories for the declaration of the COVID-19 alarm state: Age-related differences

**DOI:** 10.1007/s00426-025-02140-1

**Published:** 2025-06-05

**Authors:** Alaitz Aizpurua Sanz, Malen Migueles Seco, Iratxe Unibaso-Markaida

**Affiliations:** 1 Psychology Department, University of the Basque Country (UPV/EHU), Donostia-San Sebastian, Spain; 2 Psychology Department, International University of Isabel I (Ui1), Burgos, Spain

## Abstract

There is little research on flashbulb memories (FBM) concerning the COVID-19 pandemic, and previous findings on age-related differences in FBM are mixed. This study aims to analyse whether the recall of the declaration of the alarm state triggered by COVID-19 has the characteristics of FBM, and to examine age-related differences considering three age groups: young, middle-aged, and older adults. Typically used canonical categories were examined (e.g., where were you when you heard the news, what were you doing?) considering the specificity (amount of detail in the responses) and confidence in each response. Additionally, thoughts and emotions generated by the event were analysed. The results show that the alarm state statement meets the main characteristics of FBM; that is, participants recalled the required categories with detail, high perception of reliving, and confidence. Moreover, young people recalled the reported categories in more detail than middle-aged or older adults did. Interestingly, middle-aged people behaved similarly to older people, showing the need to consider this age group in future memory research.

**Supplementary Information:**

The online version contains supplementary material available at 10.1007/s00426-025-02140-1.

## Introduction

Flashbulb memories (FBM) are vivid and confidently held representations of how people learn about an important, surprising and consequential public event (Conway, 2013; Hirst & Phelps, 2016; Talarico, 2023). They are detailed and long-lasting memories of significant and emotional events not directly experienced (Brown & Kulik, 1977). The COVID-19 pandemic, as an unprecedented global event, provides the ideal context to explore how such memories are formed and maintained in individual and collective memory. The health crisis was of such a magnitude that on March 11, 2020, the World Health Organisation declared the COVID-19 outbreak a pandemic. This declaration prompted policies in most countries worldwide to control the spread of the virus and reduce the number of infections and deaths. One of the measures adopted was the declaration of a state of alarm, which implied the confinement of the population to their homes, the impossibility of maintaining social contacts, and the restructuring of jobs and teaching in schools and universities, as well as a situation of uncertainty and generalised fear. Three days later, on March 14, 2020, the Spanish government declared a state of alarm to address the health situation caused by COVID-19.

Does the memory of the COVID-19 alarm state declaration meet the characteristics of FBM? FBM formation is a widely studied topic, including diverse public events such as the assassination of JFK (Brown & Kulik, 1977; Yarmey & Bull, 1978) or the 9/11 terrorist attacks (e.g., Conway et al., 2009; Talarico & Rubin, 2003). Although FBM, like other emotional autobiographical memories, include central details of the event, they are characterized by capturing the context and circumstances in which individuals learned about the shocking news. Thus, FBM include personal and contextual content, such as what people were doing when they learned about the event, who informed them or how they learned about it, and their thoughts and feelings at the time they learned about the event (Castillo et al., 2024; Hirst & Phelps, 2016). Additional features of these FBM are the high level of confidence about the accuracy of these memories (Talarico, 2023) and specific recall of contextual details (e.g., the weather or how they were dressed at the time, Lanciano et al., 2018).

The study of FBM is not free of methodological and measurement problems (e.g., Coway, 2005; Thomsen & Berntsen, 2003). In their seminal paper, Brown and Kulik (1977) assessed participants'recall of the circumstances in which they learned about the JFK assassination and identified several canonical categories that characterize these memories. These categories help structure how people recall the circumstances of learning about an event. The six commonly recognized categories contributed by more than 50% of the participants in that study were referred to as the *canonical categories*. They were the following: place, ongoing activity, informant, the aftermath of the event, personal reactions to the event and the reaction of others. Although emerging from a single study, this canonical organization has been the basis for most FBM studies (Conway, 2013; Kızılöz & Tekcan, 2013; Talarico, 2023). However, these canonical categories have been extended to include other elements of the situation, such as what people were thinking when they heard the news (Kızılöz and Tekcan, 2013) or the existence of other people present (e.g., Neisser & Harsch, 1992). An additional problem of FBM is the great heterogeneity and variety of events labelled as FBM. Some studies analyse a predictable event, such as the Brexit victory in the UK (Raw et al., 2023) or the resignation of Margaret Thatcher (Cohen et al., 1994; Conway et al., 1994), events that were not surprising or unexpected as they were prolonged over time and preceded by political campaigns, demonstrations and tensions in the population. In contrast, another series of studies have focused on totally unexpected, unique and one-off events such as the 9/11 terrorist attacks (Conway et al., 2009; Talarico & Rubin, 2003), the Challenger Space Shutter disaster (Neisser & Harsch, 1992) or the assassination of relevant politicians such as the Swedish president Olof Palmer (Christianson, 1989) or JFK (Brown & Kulik, 1977; Yarmey et al., 1978). Some studies even call events of a more personal or private nature (e.g., Lanciano et al., 2018) or neutral or positive FBMs (e.g., Kraha & Boals, 2014). All these events differ in many relevant dimensions, such as level of surprise, personal involvement, emotional valence or duration of the event, although they seem to share the defining characteristics of FBM (Conway, 2005).

The main objective of this study is to analyse whether the recall of an event of worldwide impact, such as the declaration of an alarm state due to COVID-19, meets the typical characteristics of FBM. Specifically, following the criterion of Bohannon and Symons (1992), two parameters were examined: The quantity of details recalled (specificity), and the degree of confidence individuals have regarding their memories (confidence). The evaluation of the characteristics of emotional events represents an important challenge for the study of autobiographical memory in general, and for research on FBM in particular. There is broad consensus about the common questions in the study of FBM (Bohannon, 1988; Brown & Kulik, 1977; Finkenauer et al., 1998), which include date, weekday, time of the day, weather, clothes, location, informant, ongoing activity, and other significant details (e.g., who you were with when you heard about it). These canonical details surrounding the context in which the individual first learned about the event have been systematically ordered in different research measures, such as the Flashbulb Memories Checklist (Lanciano et al., 2018) used in this study, which allows us to examine more precisely the specificity of the information or the number of details provided. Furthermore, inspired by Talarico et al., (2019; see also Christianson, 1989; Kızılöz & Tekcan, 2013), we include an additional category called “ongoing thoughts” that complements the memory assessment for classical canonical details. In addition, unlike previous research which assesses global confidence about recall as a whole using phenomenological scales, in this study, we will also analyse response confidence for each reported category.

Very few studies have examined the FBM phenomenon concerning COVID-19, and these investigations focused on the participants’ feelings about the pandemic (Castillo et al., 2024), the announcement of the campus’ closure and the transition to remote learning (Xuan et al., 2024), the impact of social networks and personal involvement in the formation of false memories (Ma & Wan, 2022) or the first COVID-19 cases in different countries (Lanciano et al., 2024), not on the lockdown derived from the health crisis (for an exception with Korsakoff patients, see Herrmann et al., 2023). Lanciano et al. (2024), through a survey conducted in 11 countries, investigated FBM formation, observing that participants had detailed memories of the date and of with whom they were when they received the news (especially in China, where the pandemic originated), and partially detailed memories of the place, ongoing activities and news source. This study focused on the formation of FBM in response to the initial news of the first COVID-19 case in each participating country. In contrast, our study examines FBM from the time when the pandemic was advanced, one month after the declaration of the COVID-19 alarm state, when cases multiplied, resulting in many deaths and affected people. Governments were taking drastic measures to curb its expansion by establishing the lockdown of the population. One aspect that links all the studies of FBM conducted during the pandemic is that they do not strictly comply with one of the defining characteristics of FBM, which is the surprise of the event. The pandemic was a time-spanning event, and many of the events analyzed (e.g., school and university closures, the first cases of infected persons, or the declaration of an alarm state) were sudden in terms of the timing of their occurrence but they were anticipated by the population or they constituted logical measures to prevent the spread of the virus. We expect the declaration of the COVID-19 alarm state in Spain to meet the two typical characteristics of FBM: specificity and confidence (Bohannon & Symons, 1992; Hirst & Phelps, 2016; Lanciano et al., 2018; Talarico, 2023).

A second aim of the current study is to examine whether the participants’ age influences FBM formation. Older people generally show a decline in their ability to form and retain new memories and a worse performance than younger people in many memory tasks (for a meta-analysis, see Rhodes et al., 2019). As individuals age, they tend to remember fewer details about events (e.g., Greene & Naveh-Benjamin, 2020), leading to a more general and less vivid memory and the use of semantic knowledge and gist-based processing to complete it (Greene & Naveh-Benjamin, 2023). However, these age differences are reduced and older adults even show an advantage over younger adults when memory is assessed in everyday contexts outside the laboratory and when natural materials and/or emotional events are used (Aizpurua & Koutstaal, 2010; Badham et al., 2023; Castel, 2005). The literature on FBM is not free of inconsistent results regarding age-related effects. Some studies find no age differences (Conway et al., 2009; Davidson et al., 2006), whereas others report that older people recall fewer categories and are less specific than younger people (Cohen et al., 1994; Kensinger et al., 2006; Lanciano et al., 2024). In a recent meta-analysis, Kopp et al. (2020) examined 16 studies on FBM comparing younger (under 40) and older (over 60) adults, finding a small to moderate age-related impairment. However, to our knowledge, there are no studies contrasting FBM in three age groups. Examining these age-related differences, distinguishing between young people (19–29 years), middle-aged people (30–54 years) and older people (55–77 years) is a priority objective of the present study. Analysing the effects of age on memory is a challenge in the field of psychology because there is no clear consensus on the age ranges included in each developmental stage. However, from the point of view of human development, there is general agreement on the existence of three very distinct stages within adulthood. On the one hand, the stage known as emerging adulthood is characterised by the exploration of identity, the choice of studies and the beginning of a career (Arnett, 2000). The middle adulthood stage involves the consolidation of adult identity and the fulfilment of work and family roles (Lachman, 2004). The stage known as old or late adulthood relates to psychological ageing, which includes coping with retirement, re-evaluating the meaning of life and adapting to change and loss (Papalia et al., 2014).

When recalling autobiographical events, people not only retrieve details of the event itself, but also report event encoding variables and express more subjective aspects such as feelings and emotions (Castillo et al., 2024; Luminet, 2018). Thus, in the present investigation, we asked participants to list the emotions the alarm state declaration had generated in them. FBM literature has mainly dealt with negative events (for an exception, see Kraha & Boals, 2014, for positive events). Therefore, individuals usually rate the emotional valence of the remembered event as negative (e.g., Talarico et al., 2019). Thus, we expect the emotional reactions to the event reported by the participants in the present study to be mainly negative. Concerning the participants'age, previous studies on autobiographical memory and aging show a positivity effect: older people recall more positive content than younger people, both of past events (Aizpurua et al., 2021; Burr et al., 2020; García-Bajos et al., 2017; Schryer & Ross, 2014) and future plans and thoughts (Berntsen & Jacobsen, 2008; Cole et al., 2016; Gallo et al., 2011). Moreover, the limited work on the impact of COVID-19 shows that, although older people were the most affected and suffered the highest number of deaths, they remembered that stage positively and envisioned the future more positively than younger people (Aizpurua et al., 2021). According to the socioemotional selectivity theory (Carstensen et al., 1999), older adults deploy cognitive control mechanisms to suppress negative stimuli to enhance emotion regulation. Therefore, in the context of the present investigation, it would be expected that by activating inhibition and blocking access to negative thoughts, older adults would show a lower recall of negative emotional reactions to the alarm state than would younger adults.

In summary, in this study, we analyse whether the recall of a concrete and easily identifiable event, such as the declaration of a state of alarm in Spain, fulfils the main characteristics of FBM. Several studies have questioned the role of surprise as a necessary antecedent of FBM, showing that expected events can also trigger FBM (Coluccia et al., 2010; Curci & Luminet, 2009; Muzzulini et al., 2020). We hypothesise that, despite not being a surprising and unexpected event, being of the same nature as those analysed in other studies (e.g., Cohen et al., 1994; Conway et al., 1994; Raw et al., 2023), it fulfills most of the canonical categories identified in previous literature on FBM. We also expect older people to provide less specific details than younger adults and to have a more global recall, providing data based on familiarity and prior knowledge (e.g., Greene & Naveh-Benjamin, 2023). In alignment with the positivity literature (e.g., Burr et al., 2020; Cole et al., 2016), older people will show a greater number of positive emotions about the alarm state statement than younger people. Determining whether the performance of middle-aged people more closely resembles the recall pattern of young adults or older people is a challenge the present study attempts to address.

## Method

### Participants

A total of 112 participants were recruited in Spain through a call on the university website and social media. The young participants were mainly students of different degrees at the University of the Basque Country (UPV/EHU), and the older and middle-aged participants came from cultural groups or were undergraduate students at the University of the Basque Country (UPV/EHU) who pursued a humanities career for older people called Experience Classrooms.

Participation was voluntary, and no monetary compensation was provided. To determine the sample size, a power analysis was conducted using G*Power 3.1 (Faul et al., 2009), considering a medium effect size (*f* = 0.25) and a significance level of α = 0.05. This analysis indicated a minimum sample size of 102 participants to achieve 80% statistical power. Data collection was concluded upon reaching the 80% statistical power criterion, ensuring that the study met the minimum statistical power requirements. The sample included 49 young adults (*M* = 20.45, *SD* = 1.8; range: 19–25 years), 33 middle-aged adults (*M* = 42.76, *SD* = 6.87; range: 28–54 years), and 30 older adults (*M* = 63.53, *SD* = 5.96; range: 55–77 years). 72.3% of the sample were women. All young participants were university students, and most middle-aged (63.6%) and older adults (60%) reported having higher education.

### Materials and procedure

This study received approval from the Ethics Committee of the University of the Basque Country (UPV/EHU, ensuring compliance with APA guidelines and the Declaration of Helsinki. Participation was voluntary, and the completion of the survey presumed consent. Written informed consent was not provided because this study was conducted online through Google Forms.

The survey was disseminated through the student council, coordinators, undergraduate delegates, and the university’s website, which opened a space for studies linked to the COVID-19 pandemic. Responses were accepted from April 16 to May 12, 2020. In the form, participants were informed that the study aimed to understand how we remember personally relevant events that also have an international impact, such as the coronavirus crisis and the lockdown of the population due to the pandemic. Specifically, the instructions were: “*We are going to ask you 20 questions about your memory of where you were, what you were doing and what you felt when you learned that the government had ordered an alarm state and the entire population was to be confined to their homes. Please answer the questions as accurately and in as much detail as possible. We are also interested in your confidence in the answers to determine how sure you are that the events took place as you remember them. You can stop answering at any time.”* After each question, participants were given a space to write their answer, followed by a 7-point Likert scale (ranging from 1 *not at all confident* to 7 *totally confident*) to indicate their confidence level in their answer. Participants worked at their own pace, and there was no time pressure for the task.

Thus, 20 items were presented to the participants. All the questions were in Spanish. The first 16 open-ended questions addressed canonical categories of FBM, the next 2 items were related to encoding variables, and the last 2 items examined the phenomenological aspects of the participants’ memories. The 10 canonical categories examined were: 1) date, 2) weekday, 3) time of the day, 4) weather, 5) clothes, 6) location, 7) informant, 8) ongoing activity, 9) other significant details, and 10) ongoing thoughts. The last four of these 10 categories included more than one question. Thus, two items were presented for the Informant category: “*How did you hear about it? Describe in as much detail as possible*”, and *“Who was the first person who called or sent you a message after hearing the news?”*. For Ongoing Activity, another two items were presented: “*What were you doing when you heard the news?”* and *“What did you do immediately after you heard the news?”* Four questions were employed for the category of Other Significant Details: “*Who was the last person you spoke to before you heard the news?”, “Were you alone or accompanied by other people?” “If you were accompanied, who were you with at the time?”* and “*Who was the first person you called or sent a message to after hearing the news?”.* See Appendix 1 for a list of all the specific questions employed, presented according to their corresponding category.

Of these 10 canonical categories, the first nine derived from the Flashbulb Memories Checklist (FBMC; Lanciano et al., 2018) and correspond to the traditional categories of FBM (Bohannon, 1988; Brown & Kulik, 1977; Finkenauer et al., 1998; for a recent review, see Talarico, 2023). We added the canonical category called “ongoing thoughts”, which was inspired by Talarico et al. (2019), which included two items: “*Do you remember what you were thinking about before you heard the news?”* and “*What was your first thought?”.*

For the encoding variables (Luminet, 2018), all participants were asked whether the event was surprising and to describe how they felt about it, reporting their emotional reactions: “*Was it a surprising event for you?”* and “*How did you feel, what was your emotional reaction? Use as many adjectives as you want*”. Confidence ratings were not requested for these answers.

Finally, to assess the phenomenological aspects of their memories, participants completed two 7-point rating scales drawn from the Autobiographical Memory Questionnaire (Rubin et al., 2003). Specifically, participants rated how much they were reliving the original event: “*When I remember that moment now, I can see, hear and perceive it again in my mind,”* ranging from 1 *(Not at all clear*) to 7 *(As if it were happening now*), and how much they believe in the accuracy of their memory: “*I remember the events as they really happened and there are no contents imagined or created by my mind”,* ranging from 1 (*100% imagined*) to 7 (*100% accurate*). No confidence ratings were requested for the answers in these scales.

### Scoring

Two trained independent raters, blind to each other’s results, separately coded the participants´ responses to the open-ended questions for all the canonical categories. They employed previously established methods to investigate FBM formation (Lanciano et al., 2018). Accordingly, response specificity was scored as 2 when the participant's response was a fully detailed recall (e.g., *“I was dressed in blue sweatpants and a grey hoodie”*); a score of 1 was assigned when the response was partially detailed (e.g., sportswear); and a score of 0 was assigned when the response was missing or irrelevant to the question (e.g., *“I don't remember*” or “*I was dressed as usual*”). In the case of categories that included more than one question, the average value of each category was calculated for the analyses (See Appendix 2 for the scoring sheet employed).

Means and standard deviations of specificity and confidence levels were calculated for each of the 10 canonical categories: 1) date, 2) weekday, 3) time of the day, 4) weather, 5) clothes, 6) location, 7) informant, 8) ongoing activity, 9) ongoing thoughts, and 10) other significant details. For 25 participants (22.32% of the total sample), the two raters independently scored the specificity of each canonical category and the encoding variables’ responses with high interrater reliability (Mean* r* = 0.97; range = 0.94—1.00). Disagreements were resolved by discussion. These individual scores were averaged to obtain for each of the canonical categories a total specificity measure ranging from 0 (missing or irrelevant answer) to 2 (totally detailed recall), and a total confidence score ranging from 1 (*not at all confident*) to 7 (*completely confident*).

In addition, two encoding variables were calculated: (1) the percentage of participants for whom the event was surprising and (2) the mean and standard deviation of positive (e.g., relief, curiosity), negative (e.g., anxiety, overwhelm) and neutral (e.g., disbelief, surprise) emotions generated by the event. Russell's (2003) model was used to determine the nature of the terms used by participants to report their emotional reaction.

Finally, as phenomenological variables, the means and standard deviations were calculated for the scales evaluating the participants’ reliving of the event and their belief in the accuracy of their memories. In these cases, confidence ratings were not requested.

## Results

The results were organised under three main headings: Specificity of flashbulb memories, Confidence of flashbulb memories, Coding variables and Phenomenological variables. In each of these sections, we begin with a presentation of the analyses conducted followed by a description of the results obtained. All data were processed with JAMOVI version 2.6.25.

### Flashbulb memories’ specificity

First, a 3 (Group: young, middle-aged, older) × 10 (Category: date, weekday, time, weather, clothing, informants, location, activity, thoughts, other details) mixed ANOVA was performed, with type III correction due to the sample being non-equivalent in the number of participants in each group, with Group as the between-participants variable, and Category as the within-participants variable (see Table 1). Descriptive data such as mean, standard deviation and effect sizes (η^2^) were also calculated, and when the effects of a variable were significant, Bonferroni post-hoc analyses with alpha correction were performed. Finally, a Pearson's bivariate analysis between Age and total FBM Specificity was also performed.

**Table 1:** Mean (SD) specificity in the total sample and each age group for each category (range 0–2)

	Total	Young	Middle-aged	Older
Date	1.58 (0.83)	1.74 (0.93)	1.45 (0.71)	1.47 (0.78)
Weekday	1.94 (0.31)	1.96 (0.29)	1.97 (0.17)	1.87 (0.43)
Time	1.75 (0.61)	1.92 (0.34)	1.61 (0.75)	1.63 (0.72)
Weather	1.15 (0.54)	1.34 (0.55)	0.98 (0.54)	1.03 (0.41)
Clothes	1.24 (0.75)	1.35 (0.72)	1.27 (0.76)	1.03 (0.77)
Informants	1.71 (0.48)	1.84 (0.36)	1.74 (0.45)	1.48 (0.59)
Location	1.50 (0.52)	1.63 (0.49)	1.42 (0.56)	1.38 (0.49)
Ongoing activity	1.80 (0.41)	1.91 (0.52)	1.86 (0.36)	1.57 (0.52)
Ongoing thoughts	1.63 (0.51)	1.74 (0.61)	1.62 (0.50)	1.45 (0.61)
Other details	1.82 (0.48)	1.90 (0.39)	1.78 (0.37)	1.72 (0.39)
Total	1.59 (0.28)	1.71 (0.22)	1.54 (0.29)	1.45 (0.29)

In the total sample, the mean level of response specificity for canonical categories on the scale, ranging from 0 *(irrelevant or inexistent answer*) to 2 (*very specific answer*), was 1.59 (*SD* = 0.28), showing that participants’ recall of the context where they learned about the declaration of the alarm state was detailed.

The effects of the Group variable were significant, *F*(2, 109) = 11.08, *p* ≤ 0.001, *η*^*2*^ = 0.17. Post-hoc analysis revealed that young adults recalled more details than older adults (*p* ≤ 0.001) and middle-aged adults (*p* = 0.02), while there was no difference between the latter groups (*p* = 0.28). The effects of the Category variable were also significant, *F*(9, 981) = 28.109, *p* ≤ 0.001, *η*^*2*^ = 0.21. The Bonferroni post-hoc analysis revealed that, among the categories, the memory for ‘weekday’ was the most specific, while the memory for ‘weather’ and ‘clothes’ were the least specific. The results of all possible comparisons and their statistical indices are presented in the supplementary material (Table 4). Finally, although the Group x Category interaction was not significant, *F*(18, 981) = 0.87, *p* = 0.62, *η2* = 0.02, we found that older adults were significantly less specific than younger adults in 5 of the 10 categories, namely, ‘whether’, ‘informants’, ‘ongoing activity’, ‘ongoing thoughts’, and ‘other details’. All Bonferroni post hoc results for the Group x Category interaction can be found in Table 6 of the supplementary material.

Pearson's bivariate analyses showed that Specificity or the ability to provide specific details negatively with Age, so the older the age, the participants showed lower specificity (*r* = −0.48, *p* ≤ 0.001).

### Flashbulb memories’ confidence

First, a 3 (Group: young, middle-aged, older) × 10 (Category: date, day of the week, time, weather, clothing, informants, location, activity, thoughts, other details) mixed ANOVA was performed, with type III correction due to the sample being non-equivalent in the number of participants in each group, with Group as the between-participants variable, and Category as the within-participants variable (see Table 2). Descriptive data such as mean, standard deviation and effect sizes (η^2^) were also calculated, and when the effects of a variable were significant, Bonferroni post-hoc analyses with alpha correction were performed. Finally, Pearson correlations were also calculated to test for relationships between participant Age and FBM Confidence.

**Table 2:** Mean (SD) confidence in the total sample and each age group for each canonical category and for the total confidence (range 1–7)

	Total	Young	Middle-aged	Older
Date	5.73 (1.57)	5.47 (2.19)	5.42 (2)	4.83 (2.41)
Weekday	5.29 (2.20)	5.47 (2.19)	5.42 (2)	4.83 (2.41)
Time	4.29 (1.96)	4.51 (1.62)	4.30 (2.16)	3.90 (2.23)
Weather	4.79 (2.02)	5.10 (1.77)	4.52 (2.32)	4.57 (2.05)
Clothes	4.72 (2.62)	5.10 (2.45)	4.85 (2.61)	3.97 (2.81)
Informants	5.92 (1.30)	5.86 (1.20)	5.91 (1.43)	6.02 (1.36)
Location	6.41 (1.26)	6.71 (0.74)	6.12 (1.43)	6.23 (1.61)
Ongoing activity	6.06 (1.24)	5.98 (1.17)	6.08 (1.39)	6.17 (1.23)
Ongoing thoughts	5.99 (1.22)	5.87 (1.14)	6.21 (0.77)	5.93 (1.68)
Other details	5.54 (2.01)	5.63 (1.72)	5.70 (1.93)	5.20 (2.50)
Total	5.50 (1.01)	5.65 (0.87)	5.47 (1.06)	5.28 (1.01)

In the total sample, the mean level of confidence in the responses for details on the scale, ranging from 1 to 7, was 5.50 (*SD* = 1.01), showing that participants’ recall of the context where they learned about the declaration of the alarm state was confidently held.

The Group factor was nonsignificant, *F*(2, 109) = 1.29, *p* = 0.29, *η*^*2*^ = 0.02, revealing no significant differences between age groups. The effects of the Category variable were significant, *F*(9, 981) = 22.395, *p* ≤ 0.001, *η*^*2*^ = 0.17. Bonferroni post-hoc analysis revealed that ‘location’, ‘ongoing activity’ and ‘ongoing thougths’ were the most confidently recalled categories whereas ‘clothes’ and ‘weather’ were the categories recalled with the least confidence. The results of all possible comparisons and their statistical indices are presented in the supplementary material (Table 5). Finally, Group x Category interaction was not significant, *F*(18, 981) = 1.03, *p* = 0.42, *η2* = 0.02. However, all Bonferroni post hoc results for the Group x Category interaction can be found in Table 7 of the supplementary material.

Finally, Pearson's bivariate analyses showed that the older people were, the less confidence they had in their responses (*r* = −0.21, *p* ≤ 0.05).

### Encoding variables

In this case, descriptive data such as mean and standard deviation were calculated. In addition, a 3 (Emotion: Positive, Negative and Neutral) × 3 (Group: younger, middle-aged, older) mixed ANOVA was carried out with the emotion scores. For the phenomenological variables, one-way ANOVA was also carried out. Furthermore, Pearson correlation was calculated between Reliving and Belief in accuracy.

The declaration of the alarm state was surprising for 48.2% of the participants, with no group differences between young people (55.1%), middle-aged adults (48.5%) and older people (36.7%), χ^*2*^(4) = 5.79, *p* = 0.215.

The results of the mixed ANOVA (see Table 3) indicated that the event generated more negative (*M* = 1.87) than positive (*M* = 0.51) or neutral (*M* = 0.48) emotions, with no statistically significant differences between the last two. Group effects were significant, *F* (2, 108) = 3.358; *p* = 0.038, *η*^*2*^ = 0.06, showing that the only significant difference (*p* = 0.015) was that younger adults (*M* = 2.17) reported more negative emotions than older people (*M* = 1.37).

**Table 3:** Mean scores (SD) of emotions, reliving, and belief in the accuracy in the total sample and each age group

	Total	Young	Middle-aged	Older
Emotions (Range)	*M* (*SD*)	*M* (*SD*)	*M* (*SD*)	*M* (*SD*)
Positive (0–4)	0.51 (0.85)	0.55 (0.89)	0.33 (0.65)	0.63 (0.96)
Negative (0–6)	1.87 (1.53)	2.17 (1.66)	1.91 (1.40)	1.37 (1.35)
Neutral (0–3)	0.48 (0.68)	0.57 (0.68)	0.48 (0.76)	0.37 (0.62)
Reliving (Range 1–7)	4.71 (1.70)	4.86 (1.72)	4.55 (1.66)	4.67 (1.75)
Belief in accuracy (Range 1–7)	4.93 (1.40)	4.84 (1.36)	4.91 (1.51)	5.10 (1.37)

*Notes:* Correlation between reliving and belief in the accuracy,* r* = 0.65, *p* < 0.001. Range for Emotions shows the range of the number of emotions produced by the participants, whereas Range for Reliving and Belief in accuracy shows the range of the scores in the scales presented to the participants

### Phenomenological variables

For reliving the event (range 1–7), the overall mean was 4.71 (*SD* = 1.70), whereas, for belief in the accuracy of the memory for the event (range 1–7), it was 4.93 (*SD* = 1.40). One-way ANOVAs were performed with the scores of both scales (see Table 3). Group differences were statistically nonsignificant. Thus, regardless of the participant's age, the declaration of the alarm state was recalled as an event that was relived to a great extent, with a firm belief in the accuracy of the recall.

## Discussion

In the current research, we analysed the typical canonical categories that characterize FBM (for a recent review, see Talarico, 2023) for the recall of the declaration of the alarm state in Spain due to the COVID-19 pandemic, following the criteria of Lanciano et al. (2018) to accurately examine the specificity of participants’ memory about the event. In relation to the main objective of the present study, participants had a very vivid recollection of the event with a high level of detail, particularly in categories such as the weekday or what they were doing when they heard the news. Not all canonical categories showed a high level of specificity (Lanciano et al., 2024), as for contents like the weather, location or how they were dressed at the time of receiving the news, generic information was provided (such as the weather was nice, they were at work or they were wearing at-home clothes). Participants could calibrate confidence for the different categories and questions asked, and the highest confidence scores were obtained for the most specific and detailed responses (Pearson *r* = 0.59, *p* < 0.001 between specificity and confidence scores). This is a relevant finding, even though it is well-known that confidence in a memory is no guarantee of its accuracy (Hirst & Phelps, 2016). As expected, the rating of confidence, belief in accuracy and knowledge of the setting and circumstances were well above what would be expected from normal, everyday memory autobiographical formation (Kraha & Boals, 2014). Therefore, it can be asserted that the recall of the announcement of the declaration of the alarm state meets the main characteristics of FBM, with participants having detailed and confidently held memories about the event.

Several studies have questioned the role of surprise as a necessary antecedent to FBM, showing that expected events can also trigger FBM (Coluccia et al., 2010; Curci & Luminet, 2009; Muzzulini et al., 2020). The declaration of the COVID-19 alarm state was perceived as a logical and predictable measure and did not come as a great surprise (only 48.2% of the participants considered it a surprise). Our data are consistent with other studies on FBM and COVID-19 that analyse salient and emotional events that were also expected by the population, such as the appearance of the first contagion in the country (Lanciano et al., 2024) or the closing of university campuses and the beginning of online learning (Xuan et al., 2024). It is also consistent with studies examining events that were highly relevant and shocking to the population, but which were expected, such as the Brexit referendum victory in the UK (Raw et al., 2023) or the resignation of Margaret Thatcher (Cohen et al., 1994; Conway et al., 1994).

Regarding age differences, although there is consensus that aging is linked to impaired performance on memory tasks and a tendency to recall fewer episodic details (Greene & Naveh-Benjamin, 2023; for a meta-analysis, see Rhodes et al., 2019), the literature shows mixed results on FBM. Some studies find no major differences between young and older adults (Conway et al., 2009; Davidson et al., 2006). In contrast, other studies report that older people recall fewer aspects and less detail than younger people (Cohen et al., 1994; Kensinger et al., 2006; Lanciano et al., 2024). The lack of consensus about the findings may be due to the age disparity. For example, Kopp et al. (2020) observed moderate differences between young and old people through a meta-analysis of 16 studies on FBM. They considered young people as being younger than 40 and older people as being over 60 years old. However, considering 3 age groups allows a more accurate assessment of the impact of aging. A group of middle-aged adults, not included in the aforementioned meta-analysis (Kopp et al., 2020), may have provided clues about the differences between younger and older adults and the effects of aging. To our knowledge, the present study is the first to include a middle-aged group to systematically compare FBM formation in young, middle-aged and older adults. Our data show that young people were more specific, providing more concrete details than older participants, with no differences in confidence about their responses. As a strategy to adapt to age-related decline, older people may develop a more generic narrative style based on familiarity and activation of prior knowledge to answer questions and show more gist-based processing than younger people (e.g., Badham et al., 2023; Greene & Naveh-Benjamin, 2023). Interestingly, there were no differences between middle-aged and older people in forming FBM, either in the specificity of recall or in the confidence in responses. Thus, our study adds to previous scientific evidence indicating that an individual’s age is related to the specificity of FBM (Cohen et al., 1994; Kensinger et al., 2006; Kopp et al., 2020; Lanciano et al., 2024) and suggests that, in the current study, the profile of middle-aged adults resembles that of older people.

An additional aim of the present study was to examine the emotions generated in participants by the declaration of the alarm state, for which they were asked to report their emotional reactions to the event (see Luminet, 2018). We expected a phenomenon known as the positivity effect to emerge, with older adults showing a greater tendency than younger adults to report positive content, as seen in many situations, including post-pandemic future or future thinking experiences (Aizpurua et al., 2021). This has been interpreted as a strategy on the part of older adults to promote balance and a sense of well-being (Burr et al., 2020), even during the COVID-19 pandemic (Carstensen et al., 2020). Although in the present study, we did not observe older adults’ greater recall of positive emotions than those of younger adults, we did obtain a similar effect, with older adults providing less negative emotions than younger adults. This finding can be interpreted in terms of the socioemotional selectivity theory (Carstensen et al., 1999), which postulates that older adults deploy cognitive control mechanisms to suppress negative stimuli to enhance emotion regulation. Specifically, older adults may have used their cognitive control resources to activate inhibition and block access to negative thoughts (García-Bajos and Migueles, 2017; García-Bajos et al., 2017; Giebl et al., 2016; Marsh et al., 2019), showing less negative emotional reactions than did younger adults.

All these findings help advance the existing knowledge about the differences between autobiographical memories and FBM and the effects of aging on FBM formation. Despite its contributions, this study also has some limitations. First, it was impossible to replicate this experiment because it is a unique and unrepeatable event. Second, the retention delay was rather short, and we did not include results of a long-term evaluation of the event. We think that the critical evolution of the pandemic in terms of the number of infections and deaths, the great media pressure, the population’s obsession to gather information, and the various prolongations of the state of alarm could bias recollection, reduce the discrimination of the original event and generate a reconstructed memory. Third, the distribution of participants into three age groups in this research, although similar to other studies (e.g., Aizpurua et al., 2021; Bopp & Verhaeghen, 2005), may present methodological problems (Royston et al., 2006). The fact that the sample for this research was a convenience sample means that our participants may not be representative of the general population, reducing its generalizability. Additionally, Spain is a very social country in which the family’s and friends’ support is very relevant and out-of-home group activities are valued, so the declaration of the alarm state and its relationship rules may have had a greater impact than in other countries, and this cultural aspect may also influence age differences in the formation of FBM. For example, Lanciano et al. (2024) recently found that, when comparing results by country, the influence of age on the specificity of FBM was not homogeneous. Thus, age positively predicted FBM specificity in Denmark but negatively in Spain and Turkey, a result that also appears in our study. Finally, due to the characteristics of online data collection, it was impossible to analyse individual differences or determine whether cognitive abilities play a relevant role in FBM formation. Examining all these factors and the particularities of cognitive functioning in middle-aged adults is a challenge that future research on memory in general, and FBM formation in particular, should consider.

## Electronic supplementary material

Below is the link to the electronic supplementary material.

Supplementary file1 (PDF 236 KB)

## Data Availability

The data that support the findings of this study are openly available in DSpace at http://hdl.handle.net/10810/69330.

## Appendix 1. List of the 20 specific questions according to their corresponding canonical category.

Date:

   Q1.- What was the date you learned of the state of emergency?

Weekday:

- Q3.- What day of the week did this state of emergency proclamation occur?

Time:

      Q2.- What time was it?

Weather:

- Q4.-What was the weather like?

Clothes:

- Q8.-How were you dressed?

Informants:

- Q5.-How did you hear about it? Describe in as much detail as possible.
- Q16.-Who was the first person who called or sent you a message after hearing the news?

Location:

- Q7.-Where were you when you heard that a state of emergency had been declared?

Ongoing activity:

- Q9.-What were you doing when you heard the news?
- Q11.-What did you do immediately after you heard the news?

Ongoing thoughts:

- Q6.-Do you remember what you were thinking about before you heard the news?
- Q18.-What was your first thought?

Other details:

- Q10.-Who was the last person you spoke to before you heard the news?
- Q13.-Were you alone or accompanied by other people?
- Q14.-If you were accompanied, who were you with at the time?
- Q15.-Who was the first person you called or sent a message to after hearing the news?

Encoding variables

- Q12.-Was it a surprising event for you?
- Q17.-How did you feel, what was your emotional reaction? Use as many adjectives as you wish

## Phenomenological variables

- Q19.-When I remember that moment now, I can see, hear and perceive it again in my mind:
- 1 Not at all clear—7 As if it were happening now
- Q20.-I remember the event as it really happened, and there are no contents imagined or created by my mind: 100% imagined—100% accurate

## Appendix 2. Scoring sheet employed. For the scoring procedure, see Lanciano et al. (2018).

**Table:**

Participant #:	Question #	Specificity			Confidence (1–7)	
		Scoring			Answer	Scoring
1. Date	1	0	1	2		
2. Weekday	2	0	1	2		
3. Time	2	0	1	2		
4. Weather	4	0	1	2		
5. Clothes	8	0	1	2		
6. Informants	5 & 16	0	1	2		
7. Location	7	0	1	2		
8. Ongoing activity	9 & 11	0	1	2		
9. Ongoing thoughts	6 & 18	0	1	2		
10. Other details	10	0	1	2		
	13 & 14	0	1	2		
	15	0	1	2		
Encoding variables	12	Surprising (Yes/No)				
	17	Emotional valence (number of positive/negative/neutral emotions)				
Memory reliving	19	1 (not at all clear) – 7 (as it were happening now)				
Belief in the accuracy	20	1 (100% imagined) – 7 (100% accurate)				
