# Supplementary material for: Flashbulb memories for the declaration of the COVID-19 alarm state: Age-related differences
Source: Psychol Res. 2025 Jun 5;89(3):109. doi: 10.1007/s00426-025-02140-1 (PMC12141116; doi:10.1007/s00426-025-02140-1)
Supplement: Supplementary file 1 — Supplementary file1 (PDF 236 KB) [file 426_2025_2140_MOESM1_ESM.pdf]

Table 4.

Bonferroni Post-Hoc Comparisons and Effect sized (Cohen`s d) for Category variable in Specificity

Category 1	Category 2	Mean Difference (1-2)	*p*-value	CI 95%		Cohen`s *d*
Date	Weekday	-.38	≤.001*	-.64	-.12	.44
	Time	-.16	1.00	-.44	.11	.32
	Weather	.44	≤.001*	.13	.78	1.34
	Clothes	.34	.16	-.04	.72	.93
	Informants	-.13	1.00	-.45	.18	.39
	Location	.07	1.00	-.22	.37	.79
	Ongoing Activity	-.22	.42	-.51	.06	.32
	Ongoing thoughts	-.05	1.00	-.35	.25	.55
	Others	-.25	.24	-.54	.04	.27
Weekday	Time	.21	.01*	.02	.40	-.20
	Weather	.81	≤.001*	.63	.99	.45
	Clothes	.71	≤.001*	.47	.96	.29
	Informants	.24	.001*	.06	.43	-.13
	Location	.45	≤.001*	.28	.63	.09
	Ongoing Activity	.15	.01*	.02	.28	-.25
	Ongoing thoughts	.33	≤.001*	.14	.51	-.05
	Others	.13	.14	-.01	.27	-.26
Time	Weather	.60	≤.001*	.37	.83	.85
	Clothes	.50	≤.001*	.20	.80	.55
	Informants	.03	1.00	-.19	.25	.05
	Location	.24	.03*	.01	.47	.35
	Ongoing Activity	-.06	1.00	-.25	.13	-.09
	Ongoing thoughts	.11	1.00	-.11	.34	.17
	Others	-.08	1.00	-.28	.11	-.11
Weather	Clothes	-.09	1.00	-.35	.15	-.11
	Informants	-.57	≤.001*	-.78	-.36	-.85
	Location	-.36	≤.001*	-.58	-.14	-.52
	Ongoing Activity	-.66	≤.001*	-.84	-.48	-1.12
	Ongoing thoughts	-.48	≤.001*	-.71	-.26	-.69
	Others	-.68	≤.001*	-.87	-.49	-1.14
Clothes	Informants	-.47	≤.001*	-.74	-.20	-.57
	Location	-.26	.10	-.54	.02	-.31
	Ongoing Activity	-.56	≤.001*	-.81	-.32	-.75
	Ongoing thoughts	-.39	≤.001*	-.64	-.13	-.50
	Others	-.59	≤.001*	-.82	-.35	-.80
Informants	Location	.21	.06	-.00	.43	.32
	Ongoing Activity	-.09	1.00	-.28	.09	-.16
	Ongoing thoughts	.08	1.00	-.10	.26	.15
	Others	-.12	.16	-.25	.01	-.26
Location	Ongoing Activity	-.29	≤.001*	-.49	-.11	-.51
	Ongoing thoughts	-.12	1.00	-.35	.10	-.18
	Others	-.32	≤.001*	-.50	-.15	-.58
Ongoing Activity	Ongoing thoughts	.17	.06	-.00	.35	.32
	Others	-.02	1.00	-.16	.12	-.04
Ongoing thoughts	Others	-.19	.01*	-.37	-.02	-.36

Table 5.

Bonferroni Post-Hoc Comparisons and Effect sized (Cohen`s d) for Category variable in Confidence

Category 1	Category 2	Mean Difference (1-2)	*p*-value	CI 95%		Cohen`s *d*
Date	Weekday	-.45	1.00	-1.15	.26	.21
	Time	1.00	≤.001*	.29	1.72	.77
	Weather	.51	1.00	-.34	1.37	.44
	Clothes	.60	1.00	-.44	1.65	.38
	Informants	-.68	.17	-1.46	.09	-.12
	Location	-1.11	≤.001*	-1.87	-.36	-.43
	Ongoing Activity	-.83	.01*	-1.54	-.12	-.21
	Ongoing thoughts	-.76	.07	-1.54	.02	-.15
	Others	-.51	1.00	-1.33	.31	-.03
Weekday	Time	1.45	≤.001*	.84	2.06	.46
	Weather	.96	≤.001*	.26	1.66	.19
	Clothes	1.05	.003*	.19	1.91	.18
	Informants	-.24	1.00	-.74	.26	-.26
	Location	-.67	.001*	-1.18	-.15	-.48
	Ongoing Activity	-.38	.60	-.89	.13	-.35
	Ongoing thoughts	-.31	1.00	-.88	.25	-.29
	Others	-.06	1.00	-.63	.50	-.19
Time	Weather	-.49	1.00	-1.23	.25	-.22
	Clothes	-.40	1.00	-1.33	.53	-.15
	Informants	-1.69	≤.001*	-2.32	-1.06	-.83
	Location	-2.12	≤.001*	-2.81	-1.43	-1.00
	Ongoing Activity	-1.84	≤.001*	-2.44	-1.24	-.95
	Ongoing thoughts	-1.77	≤.001*	-2.44	-1.09	-.82
	Others	-1.52	≤.001*	-2.19	-.84	-.72
Weather	Clothes	.089	1.00	-.79	.97	.02
	Informants	-1.20	≤.001*	-1.84	-.56	-.57
	Location	-1.63	≤.001*	-2.36	-.89	-.72
	Ongoing Activity	-1.35	≤.001*	-1.95	-.75	-.68
	Ongoing thoughts	-1.28	≤.001*	-1.92	-.63	-.59
	Others	-1.03	≤.001*	-1.72	-.33	-.46
Clothes	Informants	-1.29	≤.001*	-2.11	-.46	-.46
	Location	-1.72	≤.001*	-2.55	-.89	-.66
	Ongoing Activity	-1.43	≤.001*	-2.29	-.57	-.50
	Ongoing thoughts	-1.36	≤.001*	-2.16	-.58	-.51
	Others	-1.11	≤.001*	-1.89	-.34	-.44
Informants	Location	-.43	.21	-.92	.07	-.32
	Ongoing Activity	-.15	1.00	-.62	.32	-.09
	Ongoing thoughts	-.08	1.00	-.52	.37	-.05
	Others	.17	1.00	-.27	.62	.10
Location	Ongoing Activity	.28	1.00	-.20	.77	.23
	Ongoing thoughts	.35	.61	-.12	.82	.28
	Others	.60	.005*	.09	1.11	.41
Ongoing Activity	Ongoing thoughts	.07	1.00	-.36	.49	.05
	Others	.32	1.00	-.21	.85	.17
Ongoing thoughts	Others	.25	1.00	-.26	.76	.13

Table 6.

Bonferroni Post-Hoc Comparisons for Group x Category interaction in Specificity

	Mean Difference (SE)					
	Older vs Young	*p*	Older vs Middle-Aged	*p*	Middle-Aged Vs Young	*p*
Date	-0.09 (.07)	.59	-0.10 (.08)	.56	-0.29(.18)	.37
Weekday	-0.28 (.19)	.45	0.01 (.20)	1.00	0.01(.07)	1.00
Time	-0.28 (.14)	.12	0.03 (.15)	1.00	-0.31 (.13)	.06
Weather	-0.30* (.11)	.03*	0.05 (.13)	1.00	-0.35(.11)	.009*
Clothes	-0.31 (.17)	.21	-0.23 (.18)	.62	-0.07(.17)	1.00
Informants	-.0.35*(.10)	.004*	-0.25 (.11)	.08	-0.09(.10)	1.00
Location	-0.24 (.11)	.11	-0.04 (.13)	1.00	-0.21(.11)	.22
Ongoing activity	-0.34*(.08)	≤.001*	-0.29*(.09)	.008*	-0.05(.08)	1.00
Ongoing thoughts	-0.29*(.11)	.04*	-0.17(.12)	.54	-0.12(.11)	.84
Others	-.018*(.07)	.05*	-0.06(.08)	1.00	-0.12(.07)	.28
Total	-0.26*(.06)	≤.001*	-0.08(.06)	.56	-0.17(.59)	.01*

Table 7.

Bonferroni Post-Hoc Comparisons for Group x Category interaction in Confidence

	Mean Difference (SE)					
	Older vs Young	*p*	Older vs Middle-Aged	*p*	Middle-Aged Vs Young	*p*
Date	-0.63(.51)	.64	-0.59(.55)	.87	-0.04(.49)	1.00
Weekday	-0.43(.36)	.74	-0.04(.39)	1.00	-0.38(.35)	.84
Time	-0.61(.45)	.55	-0.40(.49)	1.00	-0.21(.44)	1.00
Weather	-0.53(.47)	.76	0.05(.51)	1.00	-0.59(.45)	.59
Clothes	-1.13(.60)	.19	-0.88(.65)	.54	-0.25(.58)	1.00
Informants	0.16(.30)	1.00	0.11(.33)	1.00	0.05(.29)	1.00
Location	-0.04(.29)	.28	0.11(.31)	1.00	-0.59(.28)	.11
Ongoing activity	0.18(.29)	1.00	0.029(.31)	1.00	0.96(.28)	1.00
Ongoing thoughts	0.07(.28)	1.00	-.028(.31)	1.00	0.34(.28)	.64
Others	-0.24(.32)	1.00	-0.04(.35)	1.00	-0.19(.30)	1.00
Total	-0.36(.23)	.36	-0.18(.25)	1.00	-0.17(.22)	1.00
